# Association of Social Needs and Healthcare Utilization Among Medicare and Medicaid Beneficiaries in the Accountable Health Communities Model

**DOI:** 10.1007/s11606-022-07403-w

**Published:** 2022-02-07

**Authors:** Jennifer Holcomb, Linda Highfield, Gayla M. Ferguson, Robert O. Morgan

**Affiliations:** 1 Department of Management, Policy and Community Health, The University of Texas Health Science Center at Houston (UTHealth) School of Public Health, Houston, TX USA; 2 Sinai Urban Health Institute, Sinai Chicago, Chicago, IL USA; 3 Department of Epidemiology, Human Genetics and Environmental Sciences, The University of Texas Health Science Center at Houston (UTHealth) School of Public Health, Houston, TX USA; 4 Department of Internal Medicine, The University of Texas Health Science Center at Houston (UTHealth) John P and Katherine G McGovern Medical School, Houston, TX USA

**Keywords:** Accountable Health Communities Model, health-related social needs, healthcare utilization, screening, emergency department utilization

## Abstract

**Background:**

Integration of health-related social needs (HRSNs) data into clinical care is recognized as a driver for improving healthcare. However, few published studies on HRSNs and their impact are available. CMS sought to fill this gap through the Accountable Health Communities (AHC) Model, a national RCT of HRSN screening, referral, and navigation. Data from the AHC Model could significantly advance the field of HRSN screening and intervention in the USA.

**Objective:**

To present data from the Greater Houston AHC (GH-AHC) Model site on HRSN frequency and the association between HRSNs, sociodemographic factors, and self-reported ED utilization using a cross-sectional design. Analyses included descriptive statistics and multinomial logistic regression.

**Participants (or Patients or Subjects):**

All community-dwelling Medicare, Medicaid, or dually covered beneficiaries at participating GH-AHC clinical delivery sites were eligible.

**Main Measures:**

Self-reported ED utilization in the previous 12 months served as the outcome; demographic characteristics including race, ethnicity, age, sex, income, education level, number of people living in the household, and insurance type were treated as covariates. HRSNs included food insecurity, housing instability, transportation, difficulty paying utility bills, and interpersonal safety. Clinical delivery site type was used as the clustering variable.

**Key Results:**

Food insecurity was the most common HRSN identified (38.7%) followed by housing instability (29.0%), transportation (28.0%), and difficulty paying utility bills (26.7%). Interpersonal safety was excluded due to low prevalence. More than half of the beneficiaries (56.9%) reported at least one of the four HRSNs. After controlling for covariates, having multiple co-occurring HRSNs was strongly associated with increased risk of two or more ED visits (OR 1.8–9.47 for two to four needs, respectively; *p* < 0.001). Beneficiaries with four needs were at almost 10 times higher risk of frequent ED utilization (*p* < 0.001).

**Conclusions:**

To our knowledge, this is only the second published study to report screening data from the AHC Model. Future research focused on the impact of multiple co-occurring needs on health outcomes is warranted.

## BACKGROUND

Health-related social needs (HRSNs) are individual manifestations of social factors, such as housing instability, which impact healthcare utilization, cost, and/or health outcomes, but are not treatable by medical care^1–6^. Unmet HRSNs result in higher rates of chronic conditions, emergency department (ED) use, hospital readmissions, and no-show appointments.^7–11^ Few studies have examined multiple HRSNs through a standardized screening tool, large-scale randomized controlled trials (RCT), diverse healthcare settings, geographies, and populations.^12–14^ We analyzed data from a representative sample of community-dwelling Medicare and Medicaid beneficiaries through the national CMS Accountable Health Communities (AHC) Model.^15^ The AHC Model, launched in May 2017,^1,15^ uses the AHC screening tool to assess five HRSNs: housing instability, difficulty paying utility bills, food insecurity, transportation, and interpersonal safety. Screening is administered through various modes (i.e., paper or electronic), multiple languages, and multiple healthcare settings (e.g., clinics, EDs).^1,15,16^ Lewis et al.^17^ demonstrated concordance between the AHC screening tool and the Your Current Life Situation (YCLS) screening tool developed by Kaiser Permanente Care Management Institute (adjusted kappas range .75–.87) on food insecurity, transportation needs, and utility needs. Only housing quality had a lower kappa (.52). The authors hypothesized that wording differences in the items might lead to differences in performance between AHC and YCLS, with AHC housing items being more sensitive to identifying housing problems. The ACH screening tool was also predictive of fair or poor self-rated health (ORs 1.80 to 2.85 across needs).^17^ In AHC, beneficiaries who reported at least one HRSN and two or more ED visits in the last 12 months prior to visiting the clinical delivery site (CDS) are deemed “high risk” and randomized to receive one of two interventions, referral to community resources or referral plus patient navigation. In the first AHC Model-wide evaluation report, the relationship of multiple HRSNs and healthcare utilization was identified as a focus for future examination.^18^ No studies to date have reported on the impact of multiple HRSNs on ED utilization risk. We present data from the Greater Houston AHC (GH-AHC) Model site describing HRSNs and association with self-reported utilization.

## METHODS

### Setting and Participants

Data was collected from September 2018 through December 2020. The University of Texas Health Science Center at Houston (UTHealth) School of Public Health served as the bridge organization with 13 CDSs. CDSs included four ambulatory clinics, five hospital-based EDs, and four hospital labor and delivery (L&D) departments in the Greater Houston area, the fifth-most populous area in the USA with roughly seven million residents.^26^ The Census estimates that 42% of residents are Hispanic, 31% are White, 19% are Black, and 8% are Asian or another racial/ethnic identity in Harris County.^19^ Alongside this population growth comes increased HRSNs, exacerbating growing health inequalities in the area and nationally.^19–21^

All community-dwelling Medicare and Medicaid beneficiaries, including those dually enrolled, seeking care from one of the 13 CDSs were eligible for screening. Beneficiaries were eligible to be screened up to and including five business days before their scheduled ambulatory clinic appointment and up to and including five business days after their visit to the hospital ED. Labor and delivery beneficiaries were eligible to be screened during their hospital stay. In-person screening used a rolling kiosk with an embedded tablet in EDs and labor and delivery units as the primary mechanism, allowing for electronic patient self-screening. Telephonic screening was used for any beneficiaries who were ineligible for in-person screening (e.g., auto accident, no triage surgical cases) and ambulatory patients. A proxy (parent or guardian) answered screening questions for eligible beneficiaries under 18 years old unless otherwise determined by the CDS. Eligible beneficiaries were screened using the 10-item AHC screening tool.^1^ All consenting beneficiaries completed screening via a data collection system that was accessed via tablet for in-person screenings or computer for phone-based screenings.

### Main Measures

#### Healthcare Utilization Outcome: Self-Reported ED Visits in the Last Year

Zero visits were categorized as “0,” one visit was categorized as “1,” and two or more visits were categorized as “2.” For beneficiaries screened in the ED, the current visit was included in the total count of ED visits in the last year if the visit occurred in a hospital-based ED. Urgent care visits were excluded per the AHC screening tool. This requirement meant for beneficiaries recruited in the hospital ED, zero was not a possible outcome. Beneficiaries in ambulatory or L&D CDSs could have zero visits reported.

#### Covariates

We examined four of the five core HRSN domains: housing instability, food insecurity, transportation, and difficulty paying utility bills. Interpersonal safety data were excluded due to low prevalence. Each HRSN was dichotomized^1^ with total needs coded as 0–4. CDS type included hospital EDs, L&Ds, and ambulatory clinics which were dichotomized as hospital or outpatient. Three demographic variables reported from CDSs were used for descriptive analysis. Demographic variables in the AHC screening tool were collected as categorical data except for household size. Health insurance was collected as Medicare, Medicaid, or dual. Age of beneficiary was collected as under 18, 18 to 35, 36 to 64, or 65 and older in years. Sex for each beneficiary was female or male. In subset analyses, five additional demographic variables from the first year of the model were analyzed. Ethnicity was Hispanic/Latino/a, or of Spanish origin. Race was Black or African American; White; Asian; Native Hawaiian or other Pacific Islander; or other. Education (highest year of school completed) was less than high school degree or General Educational Development (GED), high school or GED graduate, some college, or college graduate. Annual household income from all sources was collected as under $20,000, $20,000 to $50,000, or over $50,000. Household size was collected as continuous in the AHC screening tool and dichotomized as 1 to 5 or 6 to 11 people for analysis.^22,23^ In September 2019, UTHealth switched to their own non-CMS data system. Organizations using non-CMS data systems were encouraged, but not required, to collect optional demographic questions.

### Data Analysis

We examined self-reported ED visits in the last year across four HRSNs, total number of needs, and demographic variables. Descriptive statistics summarized beneficiaries’ characteristics. Pearson correlation was used to examine prevalence of co-occurring needs. Chi-square tests compared self-reported ED visits and covariates. We conducted a clustered multinomial logistic regression with zero, one, or two or more ED visits in the last year as the outcome controlling for beneficiary characteristics, obtaining RRRs and 95% CIs. Observations were clustered within CDSs. Statistical analyses used Stata 14.0 (College Station, TX) with *α* = 0.05 for statistical significance. GH-AHC implementation was deemed quality improvement. The data analysis received approval from UTHealth’s IRB.

## RESULTS

Beneficiaries who had no screening responses or were ineligible were excluded, yielding a total sample of 15,071. Beneficiaries with a food need made up the largest group (*n* = 5830; 38.7%) compared to housing (*n* = 4373; 29.0%), transportation (*n* = 4227; 28.0%), and utility (*n* = 4024; 26.7%) need. 43.1% (*n* = 6489) of beneficiaries had zero needs, 20.2% (*n* = 3049) had one need, 16.3% (*n* = 2455) had two needs, 12.1% (*n* = 1817) had three needs, and 8.4% (*n* = 1261) had four needs. The correlation of HRSN ranged from .33 (utilities and transportation) to .4 (housing and food) for all combinations, indicating a fairly consistent strength of association between HRSNs for those with multiple needs.

### Factors Associated with Self-Reported Emergency Department Visits

Table 1 shows bivariate associations between self-reported ED visits with HRSNs and demographic factors. Over half of beneficiaries reported two or more ED visits in the last year and an HRSN (*n* = 8590; 57.0%). The number of ED visits in the last year differed significantly by total number of needs, as well as across the four individual HRSNs, CDS type, health insurance type, age, sex, ethnicity, race, education, household size, and annual household income. Compared to those with one or zero ED visits in the last year, respectively, those who had two or more ED visits were more likely to have a housing need (*n* = 5636; 34.4%), a food need (*n* = 3779; 44.0%), a transportation need (*n* = 2949; 34.3%), and a utility need (*n* = 2703; 31.5%). Those who had two or more ED visits in the last year were more likely to be dual enrollees (*n* = 862; 10.0%) and 36 to 64 years old (*n* = 2305; 26.8%) compared to those with one or zero ED visit in the last year. Over half of beneficiaries were female.

**Table 1:** Factors Associated with Self-Reported Emergency Department (ED) Visits in the Last Year Among CMS Beneficiaries in the Accountable Health Communities Model (*n* = 15,071)

Variable	0 ED visit (*n* = 894)	1 ED visit (*n* = 5587)	2 or more ED visits (*n* = 8590)	*p* Value*
Housing need				< 0.001
No	700 (78.30%)	4362 (78.07%)	5636 (65.61%)	
Yes	194 (21.70%)	1225 (21.93%)	2954 (34.39%)	
Food need				< 0.001
No	629 (70.36%)	3801 (68.03%)	4811 (56.01%)	
Yes	265 (29.64%)	1786 (31.97%)	3779 (43.99%)	
Transportation need				< 0.001
No	737 (82.44%)	4466 (79.94%)	5641 (65.67%)	
Yes	157 (17.56%)	1121 (20.06%)	2949 (34.33%)	
Utilities need				< 0.001
No	756 (84.56%)	4404 (78.83%)	5887 (68.53%)	
Yes	138 (15.44%)	1183 (21.17%)	2703 (31.47%)	
Total needs				< 0.001
0	492 (55.03%)	2902 (51.94%)	3095 (36.03%)	
1	187 (20.92%)	1114 (19.94%)	1748 (20.35%)	
2	109 (12.19%)	793 (14.19%)	1553 (18.08%)	
3	75 (8.39%)	497 (8.90%)	1245 (14.49%)	
4	31 (3.47%)	281 (5.03%)	949 (11.05%)	
Clinical delivery site type				
Hospital	640 (71.59%)	5439 (97.35%)	8350 (97.21%)	< 0.001
Outpatient	254 (28.41%)	148 (2.65%)	240 (2.79%)	
Health insurance type				
Medicare	142 (15.88%)	624 (11.17%)	1150 (13.39%)	< 0.001
Medicaid	700 (78.30%)	4682 (83.80%)	6578 (76.58%)	
Dual (Medicare + Medicaid)	52 (5.82%)	281 (5.03%)	862 (10.03%)	
Age, years				
0 to 17	202 (22.60%)	2785 (49.85%)	2577 (30.00%)	
18 to 35	348 (38.93%)	1144 (20.48%)	2122 (24.70%)	
36 to 64	123 (13.76%)	776 (13.89%)	2305 (26.83%)	
65 and older	221 (24.72%)	882 (15.79%)	1586 (18.46%)	
Sex^†,‡^(*n* = 12,759)				< 0.001
Female	553 (74.73%)	2812 (59.91%)	4587 (62.62%)	
Male	187 (25.27%)	1882 (40.09%)	2738 (37.38%)	

*Chi-square tests, ***α*** **= 0.05**

**†*n*** **with valid data**

**‡Dropped** ***n*** **= 1 for “unidentified”**

Table 2 shows subset analyses of self-reported ED visits with the five additional demographic factors from the first year of the model implementation. Compared to those with one or zero ED visit in the last year, those who had two or more ED visits were more likely to be Black or African American (*n* = 790; 59.40%), have less than a high school degree or GED (*n* = 623; 37.62%), and live in a household with an annual income less than $20,000 (*n* = 515; 74.10%). Similar ED visits were seen between household size and no trends were seen across ethnicity.

**Table 2:** Subset Analysis of Factors Associated with Self-Reported Emergency Department (ED) Visits in the Last Year Among CMS Beneficiaries in the Accountable Health Communities Model (*n* = 3128)

Variable	0 ED visit	1 ED visit	2 or more ED visits	*p* Value*
Ethnicity^†^(*n* = 2802)	**(*n* = 251)**	**(*n* = 997)**	**(*n* = 1554)**	< 0.001
Non-Hispanic	175 (69.72%)	484 (48.55%)	927 (59.65%)	
Hispanic	76 (30.28%)	513 (51.45%)	627 (40.35%)	
Race^†^ (*n* = 2314)	**(*n* = 236)**	**(*n* = 748)**	**(*n* = 1330)**	< 0.001
Black or African American	94 (39.83%)	391 (52.27%)	790 (59.40%)	
White	78 (33.05%)	130 (17.38%)	211 (15.86%)	
Asian, Native Hawaiian, or other Pacific Islander	15 (6.36%)	31 (4.14%)	34 (2.56%)	
Other^‡^	49 (20.76%)	196 (26.20%)	295 (22.18%)	
Education^†^(*n* = 2976)	**(*n* = 275)**	**(*n* = 1045)**	**(*n* = 1656)**	< 0.001
Less than high school degree or GED	55 (20.00%)	377 (36.08%)	623 (37.62%)	
High school or GED graduate	84 (30.55%)	385 (36.84%)	588 (35.51%)	
Some college	64 (23.27%)	200 (19.14%)	352 (21.26%)	
College graduate	72 (26.18%)	83 (7.94%)	93 (5.62%)	< 0.01
Household size^†^ (*n* = 3053)	**(*n* = 282)**	**(*n* = 1078)**	**(*n* = 1693)**	
1 to 5 people	253 (89.72%)	875 (81.17%)	1436 (84.82%)	
6 to 15 people	29 (10.28%)	203 (18.83%)	257 (15.18%)	
Annual household income^†^ (*n* = 1218)	**(*n* = 119)**	**(*n* = 404)**	**(*n* = 695)**	< 0.001
Under 20,000	61 (51.26%)	247 (61.14%)	515 (74.10%)	
20,000 to 50,000	33 (27.73%)	130 (32.18%)	158 (22.73%)	
Over 50,000	25 (21.01%)	27 (6.68%)	22 (3.17%)	

*Chi-square tests, *α* = 0.05

†*n* with valid data

‡Other included those selecting other, more than one racial category, or American Indian/Alaska Native

**Figure 1:**
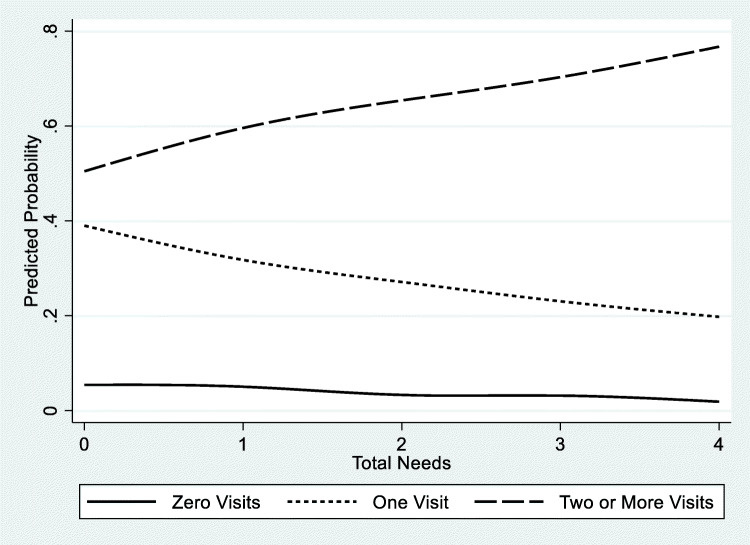
Predicted probabilities of assessing self-reported emergency department visits in the last year by zero, one, and two or more visits across total number of health-related social needs reported by CMS beneficiaries in the Accountable Health Communities (*n* = 15,071).

Table 3 shows the final multinomial logistic regression model examining the total number of needs, individual HRSNs, health insurance type, age, and sex with self-reported ED visits chosen based on log likelihood and BIC as the best-fit model. The five additional demographic variables were not included in the final model due to the reduced sample size and decreased fit. Compared to those with zero ED visit in the past year, total number of HRSNs and being male were significantly associated with a higher risk of one ED visit and two or more ED visits, while dual enrollee status was significantly associated with a higher risk of two or more ED visits. ED visit risk increased with each increase in total number of HRSNs while controlling for each individual HRSN and patient characteristics. For those with four total needs, the risk of two or more ED visits was over four times higher than zero visit and more than two times higher than one ED visit. Interestingly, compared to zero ED visit, having a food or housing need, being 18 to 35, and being 65+ years old were significantly associated with a lower risk of one ED visit, while having a food need and being 18 to 35 were significantly associated with a lower risk of two or more ED visits. To examine risk associated with the covariates, we computed predictive probabilities and average marginal effects. The predictive probability of ED visits (0, 1, and 2 or more) ranged from a mean of 0.06, 0.37, and 0.57, respectively. We examined these probabilities across the number of total needs as shown in Figure 1. The predictive probabilities in Figure 1 depict a relationship between total needs and ED visits similarly observed in Table 3. Figure 1 shows the increasing predictive probability of two or more ED visits with each increase in total number of needs. As total needs increased, the predictive probability of one ED visit decreased and zero ED visit had little change. In terms of average marginal effects, we observed that the total number of needs increased the probability of having two or more ED visits (range, 0.09 to 0.23) with small modifying effects from housing (0.01) and transportation (0.04) and negative modifying effect from a food need (− 0.04).

**Table 3:** Multinomial Logistic Regression Model Assessing Self-Reported Emergency Department (ED) Visits in the Last Year (*n* = 12,759)

Variable	Relative risk ratio (RRR), 95% confidence interval (CI)			
	1 ED visit (*n* = 5587)		2 or more ED visits (*n* = 8590)	
Housing need	0.63*	0.42–0.95	0.69	0.46–1.05
Food need	0.63*	0.44–0.90	0.57**	0.38–0.84
Transportation need	0.96	0.65–1.42	1.13	0.76–1.68
Utilities need^†^	–	–	–	–
Total needs				
0	Reference			
1	1.33	0.89–2.00	1.83**	1.27–2.62
2	2.05**	1.27–3.32	3.34***	2.26–4.94
3	2.67*	1.25–5.71	4.90***	2.25–10.68
4	4.03*	1.25–12.98	9.47***	2.72–32.96
Health insurance type				
Medicare	Reference			
Medicaid	1.08	0.55–2.12	0.96	0.46–1.99
Dual (Medicare + Medicaid)	1.15	0.86–1.54	1.46*	1.03–2.07
Age, years				
0 to 17	Reference			
18 to 35	0.25***	0.15–0.42	0.49**	0.33–0.74
36 to 64	0.55	0.27–1.09	1.35	0.69–2.64
65+	0.32**	0.15–0.70	0.53	0.25–1.14
Sex				
Female	Reference			
Male	1.40*	1.03–1.89	1.44*	1.05–1.99

*p < .05; **p < .01; ***p **< .001**

**†Omitted due to collinearity**

## DISCUSSION

Although our demographic data was limited, beneficiaries who completed social needs screening were mostly non-Hispanic, Black or African American, had low education and income levels, and lived in households with less than five people. A study by Meyer et al. similarly found most of their respondents were Hispanic, Black, low income, and living in households with 3.6 or less people.^24^ Our analysis extends their findings by comparing these demographic characteristics with ED utilization and HRSN status. We found that beneficiaries with two or more ED visits in the previous year were more likely to be Black or African American and have lower education and income levels compared to those with zero or one ED visit. All four HRSNs were related to frequent ED utilization, as was screening positive for more than one HRSN.

In our multinomial logistic regression model, after controlling for covariates (age, insurance, sex), having multiple co-occurring social needs was strongly associated with increased risk of two or more ED visits. Beneficiaries with four needs were at almost 10 times higher risk. Interestingly, food insecurity was negatively associated with frequent ED utilization when multiple needs and other domains (e.g., transportation) were included in the model. Since food insecurity was the most frequently identified need in our study, it is likely that the discriminatory power of this variable was absorbed within the multiple needs variable rather than our findings representing a true negative association between food insecurity and ED utilization. Previous studies have demonstrated an association between food insecurity and ED utilization, showing increased ED spending for food insecure households when controlling for confounders such as chronic disease status.^25^ It is important to note, however, that these previous studies of HRSN have focused on single need domain assessment, limiting their ability to discern the impact of multiple needs. In our analysis, the individual need indicators were largely non-informative in the model once the total number of needs was accounted for. Despite national initiatives to identify and address HRSN, few screening tools have included multiple need domains in a standardized way across clinical settings.^26^ Recent studies show that only 15% of providers nationwide are screening for more than one social need.^27^ More data is needed to better understand the relationship of social needs collectively and individually on ED utilization.

We also found higher rates of HRSN in the hospital rather than the outpatient setting. Much of the published literature has focused on primary care as the setting of choice for screening and intervention.^28,29^ Our data indicates that in order to reach high-risk, high-need populations, EDs should be a focal setting. Similar findings are starting to emerge.^30^ The first AHC Model evaluation report similarly found a higher percentage of beneficiaries screened in the ED were eligible for navigation (i.e., high risk) than the primary care setting (74% vs. 29%).^18^ It is well known that EDs serve a disproportionate share of underserved populations due to their inability to turn patients away who cannot pay.^31^ Research has also established that a number of patients use EDs as their primary source of care.^30–32^ Recent data from Wallace et al. support the feasibility and value of screening HRSN in EDs.^30^ Our current study adds to this emerging knowledge base by showing the frequency of positive social needs screening, ED utilization, and types of HRSN found in a large geographic area serving a diverse population across multiple health systems.

Identification of HRSN is also highly dependent on the screening tool used. Recent work by De Marchis et al.^33^ has demonstrated differences in positive identifications of housing problems obtained from the the AHC tool versus positive identification based on another widely used tool. These differences stemmed partly from different item content, e.g., housing quality versus housing stability/instability, and partly from differences in item focus, e.g., subjective stability/instability versus self-reports of actual instability. Selection of screening questions should be based on a clear understanding of the target population and the sensitivities of available tools. Further, assessing HRSNs by assessing individuals who have successfully accessed a healthcare provider may not be providing the most accurate information on the relationship between HRSN and population level health burden. Patients with lower income and educational levels tend to be less able to successfully manage their health conditions^34^. Low-income patients with unmet HRSNs have also been found to have poorer health outcomes, including increased numbers of chronic diseases.^34^ However, these patients also have well-documented difficulties accessing healthcare resources.^29,35^ Prospective studies of HRSN screening in non-healthcaresystem–based populations are needed to gain a more accurate picture of the health risks associated with increased need and the values of screening for those needs for population health. Interestingly, the AHC Model evaluation report found substantially higher rates of social needs in patients screened versus available community-level data (e.g., County Health Rankings).^18^ This is true for our GH-AHC implementation as well. Reasons for this discrepancy may include differences in constructs assessed and secondary data used to determine rates in the County Health Rankings. For example, County Health Rankings asks about budget for food, coupled with data from the USDA on food deserts, whereas AHC asks about having enough food to eat in the past month.

### Limitations

HRSNs were self-reported, making responses subject to social desirability bias and recall bias. Forty-two percent of participants completed screening through the CMS system with 58% screened using our tailored system (see Table 2). The demographic items were optional, and fewer than half of participants screened using the CMS system completed each item. Consequently, these data were missing for between 80 and 92% of the total sample, depending on the item. Further, the onset of the public health emergency in the spring of 2020 only affected participants screened using the tailored system, making the assumption of data missing at random a concern for using imputation methods across our sample. As a sensitivity test, we ran our model including race, education, ethnicity, and income on the reduced sample and findings for variables that we included in the final, full sample model were not meaningfully changed. Limiting screening eligibility to the 5-day window may have resulted in missing some beneficiaries with HRSNs. Requiring all beneficiaries to have their Medicare and Medicaid ID numbers at the time of screening meant that a large percentage of L&D beneficiaries were not able to participate due to coverage through emergency Medicaid. We lack demographic information on ineligible L&D beneficiaries with emergency Medicaid coverage and encourage caution in the interpretation of results for this population due to potential selection bias.

### Conclusion

The GH-AHC implementation addresses an important limitation of previous studies, which have typically focused on single provider settings or systems. Future research focused on the impact of multiple co-occurring needs on health outcomes is warranted.

## Data Availability

The datasets generated and/or analyzed during the current study are not publicly available due to identifying beneficiaries and clinical site information.
